# Pretreatment with VEGF(R)-inhibitors reduces interstitial fluid pressure, increases intraperitoneal chemotherapy drug penetration, and impedes tumor growth in a mouse colorectal carcinomatosis model

**DOI:** 10.18632/oncotarget.5092

**Published:** 2015-09-07

**Authors:** Félix Gremonprez, Benedicte Descamps, Andrei Izmer, Christian Vanhove, Frank Vanhaecke, Olivier De Wever, Wim Ceelen

**Affiliations:** ^1^ Department of Surgery, Ghent University Hospital, Ghent, Belgium; ^2^ Infinity (iMinds-IBiTech-MEDISIP), Department of Electronics and Information Systems, Ghent University, Ghent, Belgium; ^3^ Atomic and Mass Spectrometry, Department of Analytical Chemistry, Ghent University, Ghent, Belgium; ^4^ Department of Radiation Oncology and Experimental Cancer Research, Ghent University, Ghent, Belgium

**Keywords:** peritoneal carcinomatosis, colorectal cancer, angiogenesis, oxaliplatin

## Abstract

Cytoreductive surgery combined with intraperitoneal chemotherapy (IPC) is currently the standard treatment for selected patients with peritoneal carcinomatosis of colorectal cancer. However, especially after incomplete cytoreduction, disease progression is common and this is likely due to limited tissue penetration and efficacy of intraperitoneal cytotoxic drugs. Tumor microenvironment-targeting drugs, such as VEGF(R) and PDGFR inhibitors, can lower the heightened interstitial fluid pressure in tumors, a barrier to drug delivery. Here, we investigated whether tumor microenvironment-targeting drugs enhance the effectiveness of intraperitoneal chemotherapy. A mouse xenograft model with two large peritoneal implants of colorectal cancer cells was developed to study drug distribution and tumor physiology during intraperitoneal Oxaliplatin perfusion. Mice were treated for six days with either Placebo, Imatinib (anti-PDGFR, daily), Bevacizumab (anti-VEGF, twice) or Pazopanib (anti-PDGFR, -VEGFR; daily) followed by intraperitoneal oxaliplatin chemotherapy. Bevacizumab and Pazopanib significantly lowered interstitial fluid pressure, increased Oxaliplatin penetration (assessed by laser ablation inductively coupled plasma mass spectrometry) and delayed tumor growth of peritoneal implants (assessed by MRI). Our findings suggest that VEGF(R)-inhibition may improve the efficacy of IPC, particularly for patients for whom a complete cytoreduction might not be feasible.

## INTRODUCTION

Cytoreductive surgery with intraperitoneal chemotherapy (IPC) has been adopted by oncologic centers worldwide in the treatment of peritoneally metastasized cancer. Especially for colorectal and appendiceal carcinoma, the overall and disease-free survival of patients has improved with median overall survival reaching 33 and 130 months respectively [1, 2]. Nevertheless, the prognosis is still grave. Only a small fraction achieve five-year survival and those with an incomplete resection receive little benefit of this extensive procedure [3].

IPC utilizes a pharmacokinetic advantage conferred by the presence of the peritoneal-plasma barrier, which permits the use of high drug concentrations intraperitoneally whilst systemic absorption (and resulting toxicity) is limited [4]. Conversely, it may be assumed that the diffusion, tissue penetration, and cytotoxic effects are also limited. These aspects are mainly dependent on the molecular characteristics of each drug and the intraperitoneal pharmacokinetics have been described in previous research [5].

However, the delivery of intraperitoneal drugs is clearly also influenced by tumor type and its microenvironment. Most tumors feature a markedly raised interstitial fluid pressure (IFP) and this impedes the penetration and uptake of cytotoxic drugs [6]. Recently, experimental studies have demonstrated that reduction of the IFP by anti-VEGF(R) and/or -PDGFR therapy leads to improved delivery of systemic drugs [7]. The improved delivery combined with the disruptive effect on important tumor pathways may lead to synergistic, rather than additive, effects on tumor cell kill and growth delay. Thus far, it is unknown whether these effects could also enhance the efficacy of intraperitoneally administered drugs.

Here, we investigated whether pretreatment with anti-VEGF(R) and/or -PDGFR drugs enhances tumor penetration and increases efficacy of intraperitoneal Oxaliplatin perfusion in a mouse peritoneal carcinomatosis model. We present detailed results of intratumoral Oxaliplatin after IPC, pretreatment effect on tumor microenvironment and tumor growth delay.

## RESULTS

### Oxaliplatin inhibits HT29 cell growth *in vitro* and is comparatively more toxic in mice than in humans

**Figure 1:**
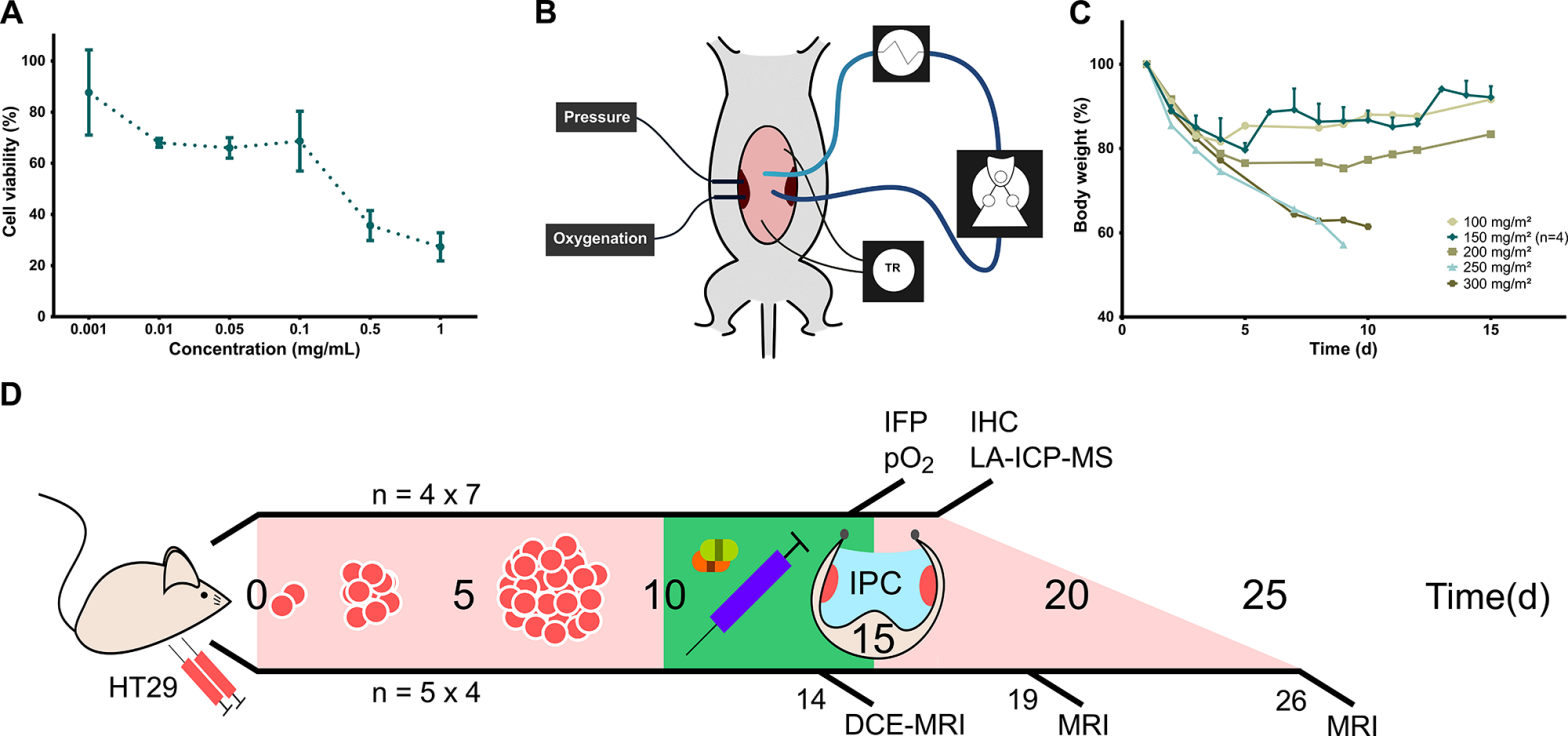
Oxaliplatin IPC model and experiment timeline **A.** Cell viability by MTT assay after application of different concentrations of Oxaliplatin with 1 h exposure (mean, standard deviation). **B.** Schematic representation of experimental set-up for Oxaliplatin IPC in mice. The perfusate flows through silicone tubing past a peristaltic roller pump and a heat exchanger. Temperature is recorded continuously and maintained around 37°C. Tumor IFP and oxygenation are monitored *in vivo* by intratumoral probes. **C.** Body weight decrease after Oxaliplatin IPC at increasing doses. IPC at 150 mg/m^2^ was repeated in three more mice to confirm the maximum tolerated dose. (Single values; 150 mg/m^2^: mean, standard deviation). **D.** Timeline of IPC experiments. The upper line shows the Oxaliplatin tumor penetration experiment with tumors resected immediately after IPC for LA-ICP-MS mapping. The lower line shows the tumor growth delay experiment in which mice underwent sequential MRI scans. *N* indicates the number of mice.

To determine cancer cell line susceptibility to Oxaliplatin, an MTT (3-(4,5-dimethylthiazol-2-yl)-2,5-diphenyltetrazolium) assay was performed (Figure 1A). The IC50 in HT29 cells after 1 h exposure was estimated at 0.343 mg/mL (95% CI 0.069 to 1.707 mg/mL). *In vivo* toxicity was evaluated by performing IPC with increasing doses of Oxaliplatin, starting at approximately 1/4^th^ of the clinical dose (100 mg/m^2^) (Figure 1B, 1C). Major toxicity and weight loss were noted in mice receiving 250 – 300 mg/m^2^ of Oxaliplatin and euthanasia was required. Necropsy uncovered no plausible surgical complications as the cause. At 200 mg/m^2^, initial dehydration, reduced activity, and food intake were noted. Weight loss and recuperation time were considered excessive. At 150 mg/m^2^ and lower doses, no major toxicity was noted and mice recovered most lost weight within two weeks. The experiment was repeated at 150 mg/m^2^ in three mice with similar results. No further toxicity or mortality due to Oxaliplatin was observed during the experiment.

### VEGF inhibition affects tumor IFP, oxygenation, and vascularity, but has no impact on size or proliferation index of HT29 xenografts

**Figure 2:**
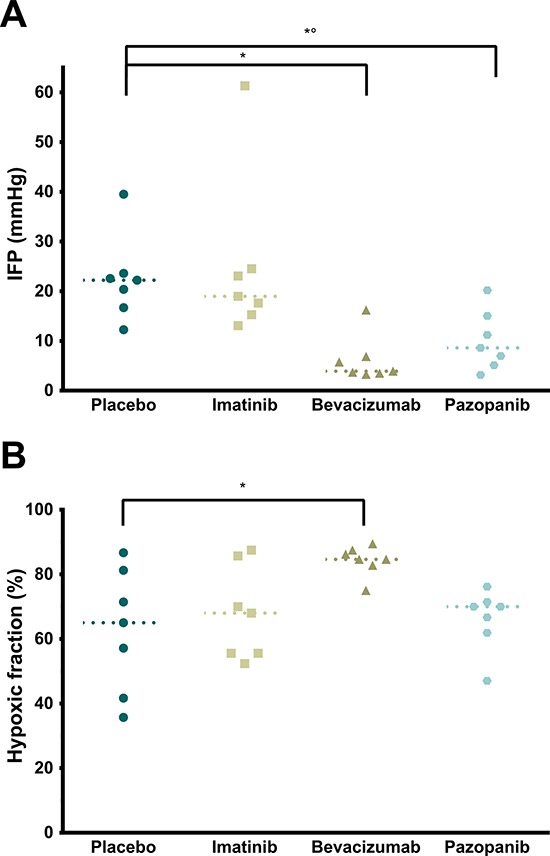
*In vivo* tumor measurements **A.** Intratumoral IFP relative to atmosphere (*p* = 0.0008, single values, median). * Placebo vs. Bevacizumab (*p* = 0.0028). *° Placebo vs. Pazopanib (*p* = 0.0407). **B.** Tumor hypoxic fraction (% < 5 mmHg pO2; *p* = 0.0257, single values, median). * Placebo vs. Bevacizumab (*p* = 0.0279).

In the first experimental series, IPC was performed in mice with two large peritoneal tumor nodules after pretreatment with either Placebo, Imatinib, Pazopanib, or Bevacizumab (Figure 1D). Intraoperatively measured tumor IFP was significantly lower in the Bevacizumab and Pazopanib groups (Figure 2A, *p* = 0.0008). Imatinib did not differ from Placebo. All tumors had low values of oxygenation. However, the hypoxic fraction was significantly increased in the Bevacizumab group (Figure 2B, *p* = 0.0257). No statistical differences were detected between the other groups. No toxicity due to pretreatment was noted and mice appeared in good general condition.

Immediately after IPC, mice were euthanized and samples collected. Macroscopically, the tumors formed irregular large nodules averaging 124.85 mm^3^. No significant difference in size was found between the four groups (data not shown, *p* = 0.1656). Tumors grew invasive in the muscular abdominal wall and formed nodules directed towards the peritoneal cavity. A few tumors broke through the external layers of the abdominal wall and showed partial invasion of the skin, but without ulceration. Rarely, adhesion to the bowel wall was noted. In the Bevacizumab group, some tumors contained small central hematomas.

**Figure 3:**
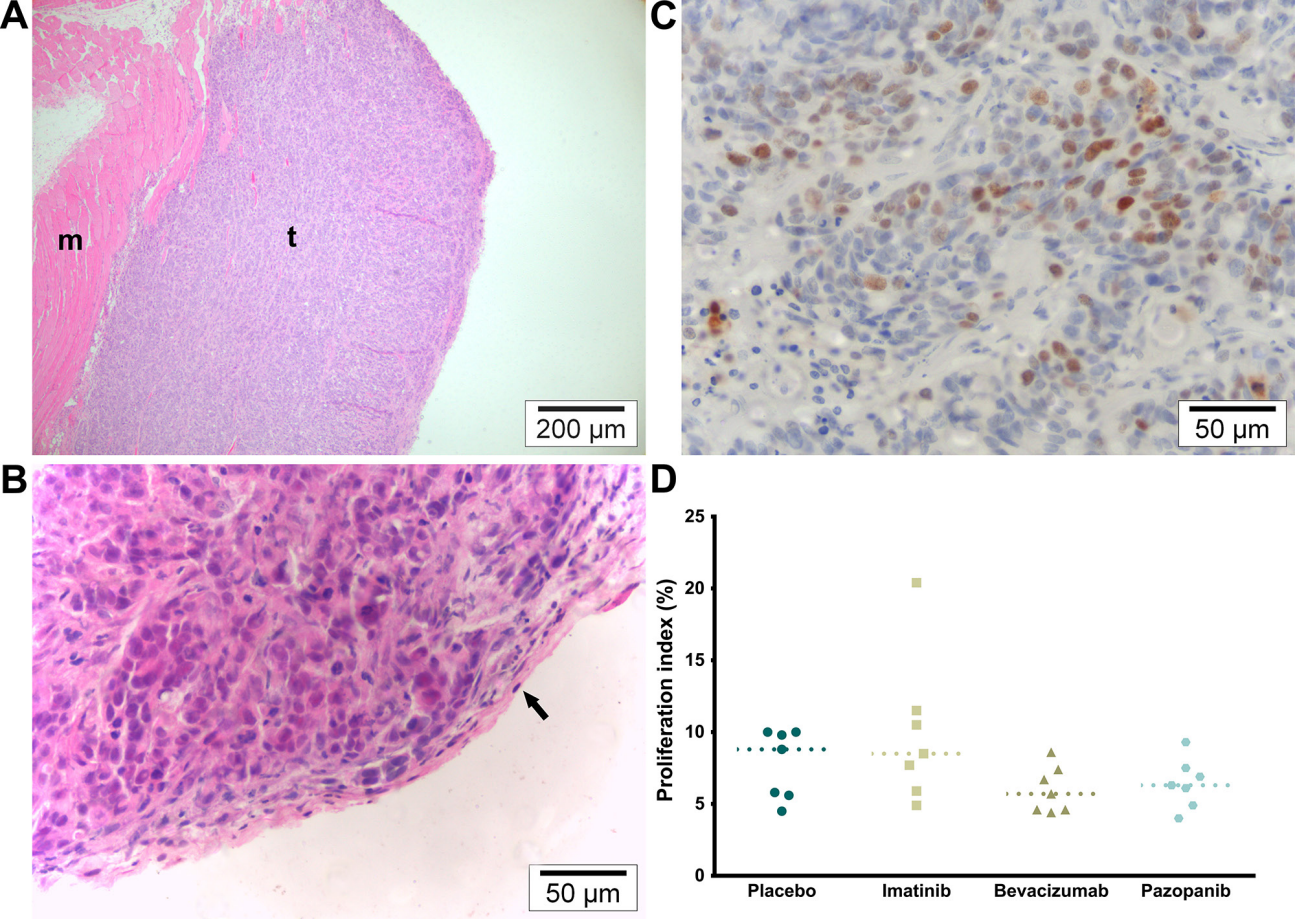
Microscopy overview and proliferation **A.** H&E (100×). Overview of representative tumor section. Tumors were sectioned according to their orientation so that each section showed both the peritoneal and the muscular border. M indicates muscle and *t* tumor tissue. **B.** H&E (400×). Detail of tumor in which the mesothelium can still be identified along the peritoneal border (arrow). **C.** KI-67 (400×). Average hot spot of tumor cell proliferation. **D.** Proliferation index (% KI67+ nuclei; *p* = 0.1482, single values, median).

**Figure 4:**
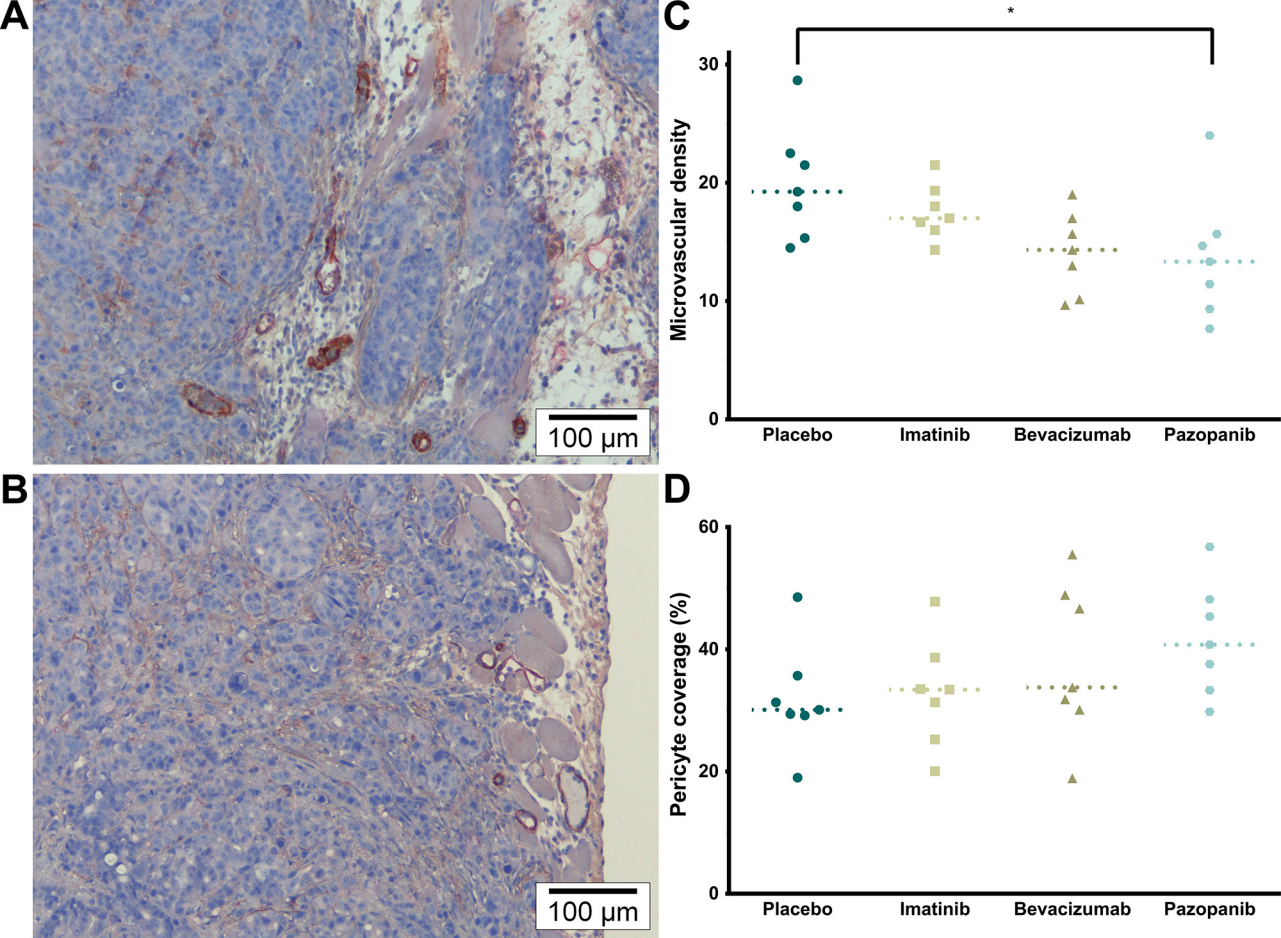
Tumor vascularity **A.** α-SMA (brown) & CD105 (red) (200×). Representative image of MVD and Pericyte coverage of the Placebo and Imatinib groups. **B.** α-SMA & CD105 (200×). Representative image of the Bevacizumab and Pazopanib groups, mainly showing reduced vascularity. **C.** Microvascular density (CD105+ vessels; *p* = 0.0323, single values, median). * Placebo vs. Pazopanib (*p* = 0.0424). **D.** Pericyte coverage (% α-SMA+ vessels; *p* = 0.2446, single values, median).

On H&E staining (Figure 3A, 3B), tumor cells were invasive in the submesothelial and muscular layers of the peritoneal wall. The mesothelium was absent from the peritoneal tumor border in most samples, except around the edges, but mice fibroblasts were visible throughout the tumor. No inflammatory response (leukocyte infiltration) could be observed in the athymic mice model. However, some central necrosis was present in the larger tumors, particularly in the Bevacizumab and Pazopanib groups. The proliferation index (Ki-67) did not differ between treatment groups (Figure 3C, 3D; *p* = 0.1482). On CD105 / α-SMA slides, there was a significant difference in the microvascular density (Figure 4; *p* = 0.0323). Mice receiving Pazopanib had a lower average number of vessels per medium power field (CD105+, 200x, *p* = 0.0424) compared to Placebo. A similar trend was identified in the Bevacizumab group (*p* = 0.0780). Pericyte coverage analysis (% α-SMA+ vessels) did not reach significance (*p* = 0.2446) and values had a large variance. These findings suggest some vascular regression in the Bevacizumab and Pazopanib groups, with little indication of increased normalization.

### Oxaliplatin concentration is greater in tumors with low IFP

**Figure 5:**
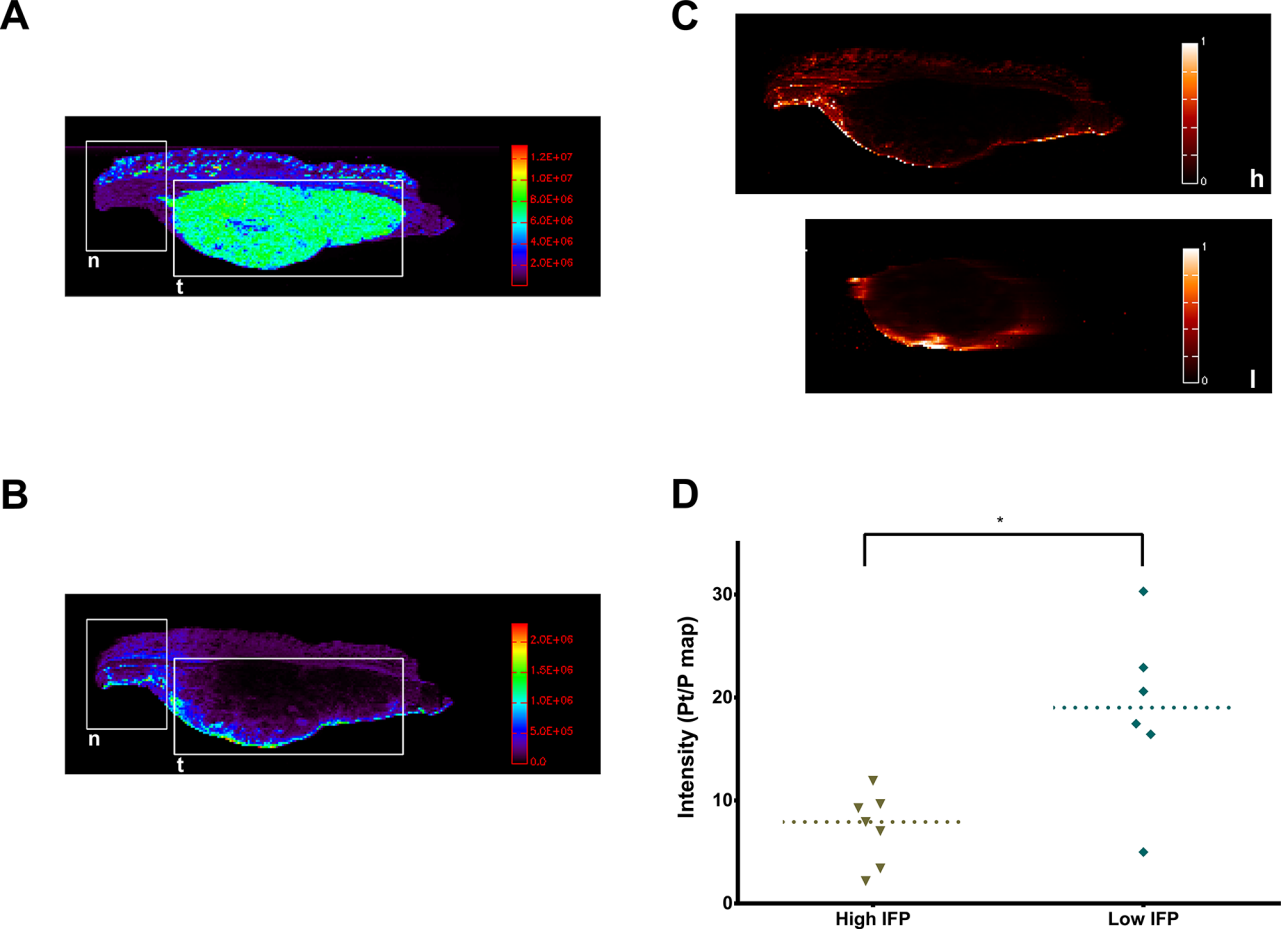
Tumor oxaliplatin distribution **A.** LA-ICP-MS color map of tumor *P* distribution (*n* = normal tissue, *t* = tumor). **B.** LA-ICP-MS color map of tumor Pt (of Oxaliplatin) distribution (*n* = normal tissue, *t* = tumor). **C.** Pt/P maps of a tumor with high IFP (h) and one with low IFP (l). **D.** Average Pt/P intensity in the tumor peritoneal border region (*p* = 0.0221, single values, median).

With laser ablation inductively-coupled plasma mass spectrometry (LA-ICP-MS), color maps were created of the Platinum (Pt) and Phosphorus (P) distribution in tumor sections. These were then combined in Pt/P ratio maps to compare Pt concentration across samples. In the peritoneal border area (up to 1.68 mm), an increased Pt/P ratio was detected in tumors with a low IFP (Bevacizumab and Pazopanib) compared to those with a high IFP (Placebo and Imatinib) (Figure 5; *p* = 0.0221, *n* = 15). Differences in tumor centers could not be assessed because of irregular tumor shapes and sizes. In those sections containing both normal tissue and tumor, the Pt maps showed a deeper penetration and a higher concentration of Pt in normal tissue compared to tumor.

### Pretreatment with VEGF-inhibitors increases tumor growth delay after IPC

**Figure 6:**
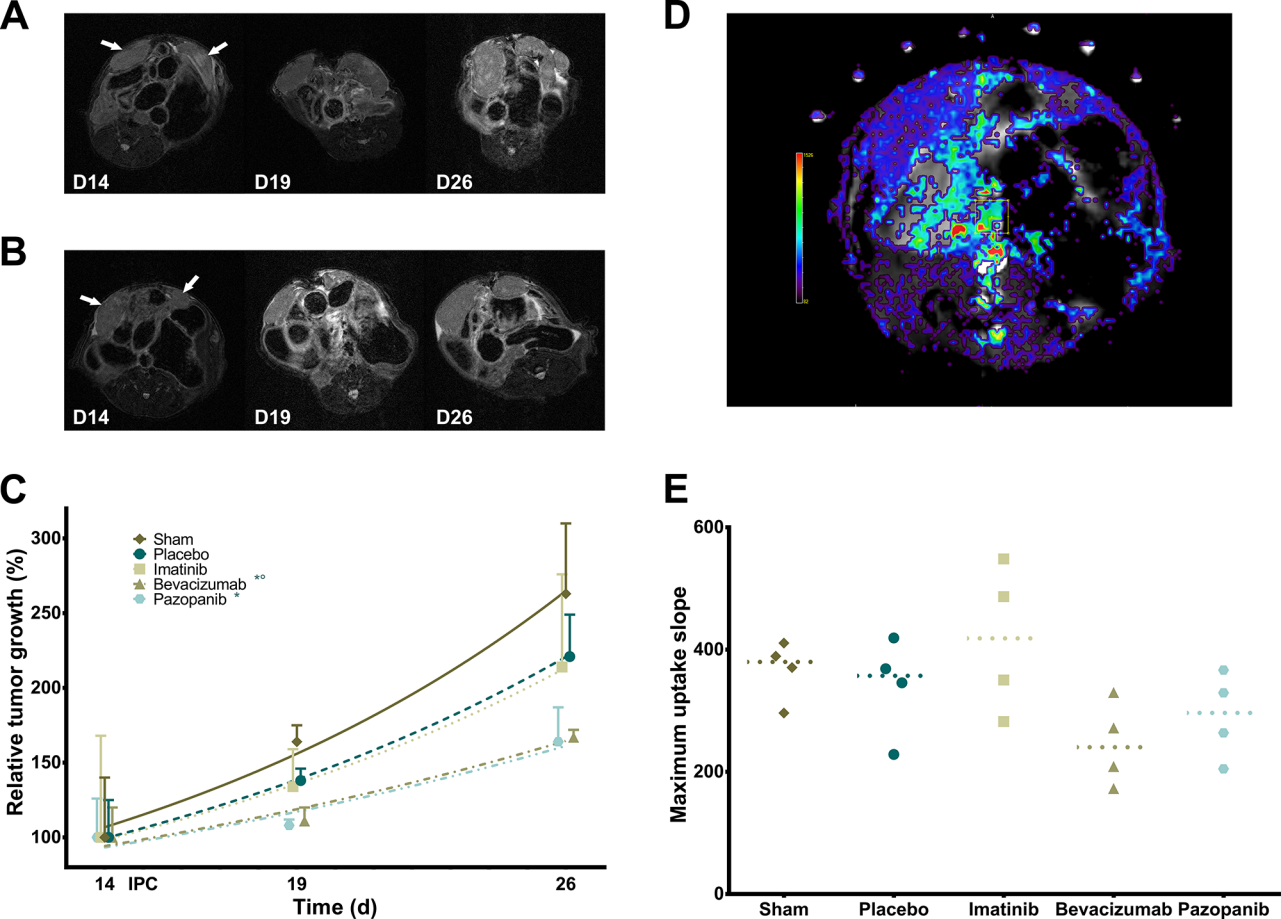
Tumor growth delay and vascularity **A.** Serial MRI images of mouse abdomen with bilateral tumor nodules (Placebo) as indicated by arrows. **B.** Serial MRI images (Bevacizumab). **C.** Relative tumor growth delay after IPC (exponential growth curves; *p* = 0.0006, mean, standard deviation). * Sham vs. Pazopanib (*p* < 0.05). *° Sham, Placebo vs. Bevacizumab (*p* < 0.05). **D.** Color overlay image of DCE-MRI analysis of maximum uptake slope. **E.** Tumor maximum uptake slope (*p* = 0.0890, single values, median).

In the second experimental series, tumor growth delay was assessed on sequential MRI scans in four groups as described previously and with an added Sham (no Oxaliplatin) control group. Relative tumor growth differed significantly between groups (Figure 6A, 6B; *p* = 0.0006). Both Bevacizumab and Pazopanib groups had slower tumor growth than the Sham group, but only Bevacizumab reached statistical significance compared to Placebo. Tumor doubling time was Sham 9.157 (95% CI; 6.687 to 14.52), Placebo 10.43 (95% CI; 8.123 to 14.56), Imatinib 10.75 (95% CI; 6.172 to 41.51), Bevacizumab 14.90 (95% CI; 11.35 to 21.68), and Pazopanib 15.20 days (95% CI; 10.28 to 29.18). Additionally, DCE-MRI was performed to assess tumor vascularity *in vivo*. Curve analysis of maximum uptake slope did not reach statistical significance (Figure 6D, 6E; *p* = 0.089). However, a trend could be observed of decreased values for the Bevacizumab and Pazopanib groups, corresponding to the IHC results.

## DISCUSSION

Here, we demonstrate for the first time that anti-VEGF(R) treatment lowers IFP in peritoneal CRC xenografts, that this facilitates the diffusion and penetration of intraperitoneal Oxaliplatin, and that this results in greater tumor growth delay. Complications in this novel mouse IPC model were successfully managed by slightly reducing the Oxaliplatin dose to 150 mg/m^2^ and changing the perfusion fluid from glucose 5% to saline. While contrary to manufacturer's instructions, several experimental and clinical reports confirm that Oxaliplatin is sufficiently stable for IPC in a chloride-containing solution when it is diluted shortly before perfusion [8, 9]. Oxaliplatin degrades into its active metabolite [Pt(dach)Cl2] in the presence of chloride at a rate of less than 20% in 120 min. Notably, the large nodule mouse model in this study differs from most peritoneal carcinomatosis models (IP injection of cancer cells) in that specific size-dependent aspects of tumor biology such as oxygenation, perfusion, and IFP may be evaluated more accurately. The tumors are consistent in size, IFP, and macroscopic aspect with intraperitoneal nodules of human CRC (completeness of cytoreduction score CC-2) [10, 11]. Moreover, this model allows the use of advanced *in vivo* (intraoperative measurements, MRI) and *ex vivo* (LA-ICP-MS) techniques to be used.

LA-ICP-MS confirms the hypothesis that the uptake of Oxaliplatin in tumors is reduced compared to normal tissue and that by decreasing the IFP, the diffusion of Oxaliplatin can be improved. The IFP of normal tissue is reported as −1 to −3 mmHg and this supports the transcapillary exchange of solutes [12, 13]. In tumors, the high IFP impedes this transport and likewise also functions as a barrier to drug uptake for IPC [14]. Even though tumors are in direct contact with the cytotoxic solution, it is likely that only a minor part of the total drug dose reaches its target. For small molecules, the large surface area of the peritoneum assures a quick drug uptake mainly through healthy tissues into the portal and systemic circulation. The low IFP samples have more Oxaliplatin in the border region, but still very little in the tumor center. Previous attempts were made to increase cytotoxic drug uptake after IPC, mostly by changing IPC parameters such as temperature, perfusate or pressure [15]. Unfortunately, few of these studies analyzed tumor samples.

It is thought that antiangiogenic therapy transiently normalizes tumor vessels; e.g. decreases leakiness and IFP, prunes small abnormal vessels, and increases tissue perfusion as well as oxygenation [16]. However, this effect appears very dependent on dose and timing of therapy. Thus far, clinical evidence has been limited. Nevertheless, neoadjuvant Bevacizumab has been shown to normalize tumor vasculature in rectal cancer [17]. Here, both Bevacizumab and Pazopanib lower the IFP as expected, but more hypoxia is noted in the former group. Microvascular density is decreased, though pericyte coverage appears unaffected. DCE-MRI reveals a trend of reduced maximum slope and this indicates antiangiogenic effect, but does not correspond directly with a single physiological parameter, rather it reflects a combination of vascular density and permeability [18]. Jain et al. describe the normalization window of antiangiogenic therapy as very brief; i.e. 6 days in mice and up to a month in man [16]. Furthermore, Tailor et al. state the possibility that normalization of oxygenation, perfusion or pressure are not necessarily concurrent [19]. These findings touch on the issue of continuous treatment with antiangiogenic therapy which is likely not ideal for every cancer and every drug. Research concerning optimal treatment windows could greatly impact current patient care [20].

Imatinib is unable to lower the IFP in HT29 tumors and subsequently does not differ from Placebo in any of the tests. HT-29 lacks PDGFR, but is reported to express PDGF and to induce the expression of PDGF in neighboring stromal cells [21]. Unlike Bevacizumab, which is quite specific for human VEGF-A, Imatinib targets both human and mice PDGFRβ [22, 23]. PDGFRβ-inhibition is presumed to lower the IFP by reducing excessive matrix density caused by fibroblast proliferation and contractility [13]. Its role in vascular normalization is more ambiguous; both antiangiogenic effects and inhibition of pericytes have been reported. Pazopanib also targets PDGFR, but this activity cannot be separately evaluated from its VEGFR-inhibition in these experiments. Possibly, PDGF(R)-targeting drugs could be more successful in a different cancer model or treatment regimen [12, 13]. The PDGF(R) pathway plays an important role in the tumor microenvironment and should be included in future research [24].

Interestingly, Oxaliplatin itself barely affects tumor growth in our experimental model. LA-ICP-MS reveals only limited tumor penetration of Oxaliplatin. Conversely, Klaver et al. demonstrated a clear impact of hyperthermic IPC (HIPEC) on survival in rats, but this after complete cytoreduction [25]. Prodige 7 (NCT00769405), an ongoing randomized controlled trial, will hopefully provide an answer on whether the addition of HIPEC to cytoreductive surgery improves survival. Presumably, IPC affects only small nodules and free floating cancer cells. Most clinical studies identify the completeness of cytoreduction as a clear prognostic indicator, with little benefit of cytoreductive surgery and HIPEC for patients with a suboptimal resection [3, 26]. Additional treatment such as neoadjuvant therapy, perhaps with VEGF(R)-inhibition, may increase resectability and improve tumor susceptibility to IPC for this subgroup of patients. The increased growth delay we observed is likely a combination of increased Oxaliplatin cytotoxicity in the peritoneal border and antiangiogenic growth inhibition. However, VEGF(R)-inhibitors are usually not very effective on their own [27, 28]. Pretreatment does not decrease tumor size at the time of IPC and there is no change in cell proliferation, but some central necrosis is present. An alternative theory supporting the combination of cytotoxic and antiangiogenic drugs presumes that there may be enhanced retention of drugs by decreased vascular clearance, which could be useful for IP therapy [22].

Importantly, the morbidity of added antiangiogenic therapy cannot be evaluated in a xenograft experiment. Bevacizumab has previously been linked to surgical and non-surgical complications and is commonly discontinued in the weeks leading up to surgery (4–8 weeks). Pazopanib may cause similar toxicity but has the advantage of a much shorter half-life (30.9 h). Nevertheless, several studies report that the possible additional morbidity of neoadjuvant VEGF(R)-inhibitors is not prohibitive, even for complex surgery [29, 30]. Moreover, Starlinger et al. demonstrated in a prospective trial that six weeks after therapy cessation, VEGF was still effectively blocked by Bevacizumab without hindering wound-healing [31].

There are some limitations to this study. First, no quantitative LA-ICP-MS mapping could be performed. Other methods that determine drug concentration in whole tissue samples would likely not have been sufficiently sensitive to detect the changes in Oxaliplatin penetration. Recent X-Ray Fluorescence pilot studies have successfully provided a detailed analysis in a similar experimental set-up (unpublished results). Future clinical trials in this center will employ either quantitative LA-ICP-MS or X-Ray Fluorescence. Secondly, no pharmacokinetic models could be fitted to the DCE-MRI data mainly due to breathing-related artefacts. Therefore, only semi-quantitative curve analysis was possible [18]. A different experimental set-up might reveal more therapy effect with DCE-MRI. Lastly, tumor samples could not be evaluated for cytotoxicity after IPC. For Oxaliplatin analysis, samples had to be resected immediately. Future experiments could examine tumors 24 to 72 h after IPC for the detection of apoptosis, DNA strand breaks, and Oxaliplatin-DNA adducts. To our knowledge, no antibody has been developed for the latter, but several exist for Cisplatin-DNA adducts [32].

In conclusion, our findings suggest that neoadjuvant therapy with VEGF(R)-inhibitors may improve the efficacy of IPC, especially for patients for whom a complete cytoreduction might not be feasible. It remains for clinical trials to determine optimal dosage and especially treatment window to achieve the desired effect. Likewise, morbidity should still be evaluated comprehensively.

## MATERIALS AND METHODS

All animal experiments were approved by the Animal Ethics Committee of the Faculty of Medicine, Ghent University, and were performed according to Belgian and European legislature on animal welfare. Mice were fed ad libitum and kept under standard conditions. All procedures were performed under general anesthesia (IsoFlo, Abbott, Belgium) and analgesia (Ketoprofen, 5 mg/kg; Buprenorphin, 0.05 mg/kg). Mice were evaluated daily for pain or discomfort and were given further analgesia up to two days post-operatively. All procedures on animals were performed in a laminated flow cabinet and standard cytotoxic drug safety precautions were applied.

### Cancer cell line

HT-29, a human colon cancer cell-line, was obtained from the ATCC. The cells were cultured at 37°C in 5% CO_2_ humidified atmosphere in McCoy's SA medium (Invitrogen, 26600-023) with Fungizone (Bristol Myers Squibb B.V, 3440 AM Woerden), 10% fetal bovine serum (Greiner Bio One, 758093), and penicillin-streptomycin (Invitrogen, 15070-063). The HT29 cells were authenticated by STR DNA profiling (ATCC, 25/02/14).

An MTT assay as described by De Smet et al. was performed to evaluate Oxaliplatin cytotoxicity *in vitro* [33]. Briefly, cells were seeded in 96-well plates at a concentration of 8 × 10^4^ cells/ml. After 24 h, 20 μL medium is replaced by 20 μL of drug solution. Oxaliplatin concentrations of 100 μg/mL, 50 μg/mL, 20 μg/mL, 10 μg/mL, 5 μg/mL, and 1 μg/mL were assessed. Eight wells per concentration were used and all experiments were repeated (3x). Next, the plates were incubated for 60 min at 37°C and 10% CO_2_. Then, the oxaliplatin solution was replaced by 200 μL fresh medium. Afterwards, the cells were incubated for 24 h at 37°C and 10% CO_2_. Finally, the cytotoxicity of the formulations was evaluated via MTT assay and measured by assessing the optical density using an ELISA-plate reader (Paradigm Detection Platform, Beckman Coulter, Suarleé, Belgium).

### Xenograft model

1.5 × 10^6^ HT-29 cells suspended in 40 μL Matrigel (Corning BV, Amsterdam, the Netherlands) were injected subperitoneally in the right and left side of the anterolateral abdominal cavity of nude athymic mice (8 WO male Foxn1nu, Harlan, Horst, the Netherlands). The peritoneal layer covering the gelled nodules was superficially incised to promote intraabdominal expansion (Supplementary Figure S1). The abdomen was closed in two layers with PDS 6/0 (Ethicon, Johnson & Johnson Intl., Brussels, Belgium).

### Intraperitoneal chemotherapy procedure

The intraperitoneal perfusion circuit was constructed out of silicon tubing (Pumpsil^®^, Watson-Marlow, Zwijnaarde, Belgium), a peristaltic roller pump (520 U, Watson-Marlow, Zwijnaarde, Belgium), and a heat exchanger (M3 Lauda, Lauda-Brinkman, NJ, USA) (Figure 1B). Anesthetized mice were placed on a heating pad and a midline laparotomy was performed. The edges of the abdominal wall were sutured with PDS 6/0 to a metal ring in a method similar to the open (coliseum) IPC technique. Oxaliplatin (100–300 mg/m^2^) was added to 2 L/m^2^ saline (NaCl 0.9%) and briefly circulated and preheated in a closed circuit. Perforated inflow and outflow catheters with attached temperature probes were placed in the abdomen and the perfusate was then circulated for exactly 60 min. Temperature was continuously measured and registered by E-Val^®^ 2.10 Software (ELLAB^®^, Roedovre, Denmark). Perfusion rate and heat exchanger temperature were adjusted intermittently by small increments to maintain the intra-abdominal temperature between 36°C and 38°C. Special care was taken to ensure that both tumor nodules were fully submerged and no perfusate was spilled. After 60 min, the perfusate was evacuated by suction. The abdomen was closed in two layers with PDS 6/0.

### Maximum tolerated dose

IPC with Oxaliplatin in increasing doses (100–300 mg/m^2^) was performed in five mice. Mice received pain relief (see above) and were euthanized in case of clear morbidity. The starting dose was defined as 1/4^th^ of that in humans relative to body surface area. The maximum tolerated dose (MTD) was defined as the highest non-lethal dose without serious adverse effects (bodyweight decrease <10% after 2 weeks) [33]. IPC with the same dose was then repeated in three more mice to confirm the MTD.

### Oxaliplatin tumor penetration

#### Pretreatment

Mice were divided into four groups of seven animals, each receiving a different pretreatment from day 10 to 15 after HT29 implantation. The first group, Placebo, received either daily oral gavage of 0.15 mL PBS or IP injections of 0.15 mL saline solution on day 10 and 14. The second, Imatinib, received daily oral gavage of 50 mg/kg Imatinib mesylate (Glivec^®^, Novartis; anti-PDGFR) in PBS. The third, Bevacizumab, was injected on day 10 and 14 with 5 mg/kg Bevacizumab (Avastin^®^, Roche; anti-VEGF (human)). The fourth, Pazopanib, received daily oral gavage of 100 mg/kg Pazopanib hydrochloride (Votrient^®^, GSK; anti-VEGFR, -PDGFR) in distilled water (0.5% HMPC, 0.1% tween 80).

#### Intraperitoneal chemotherapy

IPC was performed on day 15 (after the last pretreatment dose) with 150 mg/m^2^ Oxaliplatin for 60′ at 36–38°C as described previously. Immediately after IPC, mice were euthanized by intracardiac puncture and exsanguination. Blood and perfusate were preserved. Left tumor nodules were excised and measured in three dimensions with electronic calipers.

#### Intraoperative measurements

Tumor IFP was measured using a fine (0.36 mm) glass fiber based on the Fabry Perot interferometer (Samba Preclin^®^, Samba Sensors, Bioseb, Vitrolles, France) according to the method described by Ozerdem [34]. Briefly, the probe is placed in a perforated 30-gauge catheter filled with gel (Duratears^®^, Alcon). The catheter is then carefully introduced along a premade needle track into the center of the right tumor nodule. Tumor IFP (relative to the atmosphere, mmHg) was recorded for 5 to 8′ after achieving a stable value.

Tumor oxygenation was measured throughout the nodule using a single fluorescence based fiberoptic pO2 ‘Bare-Fibre’ sensor (OxyLite^®^, Oxford Optronix, Oxford, UK) in a method similar to Ceelen et al [35]. In short, the probe was fixed to a micromanipulator and guided through the tumor along premade needle tracks. Measurements were made every 200 μm for up to 30 data points. Data was collected with Powerlab/8sp (ADInstruments, Oxford, UK) and the hypoxic fraction (% <5 mmHg pO2) was calculated.

#### Tumor samples and histology

Tumors were placed for 24 h in formaldehyde 4% and then conserved in PBS until processed and embedded in paraffin. Sample slices (5 μm) were cut by microtome for microscopic analysis (ColorView I, BX43F, Olympus, Tokyo, Japan). Standard H&E and Ki-67 staining were carried out for each tumor. Tumor regions were identified on H&E and visually analyzed for invasion, necrosis and uncommon features. Proliferation index (Ki-67) was assessed by hot spot analysis (up to 6/slide) and quantified using ImmunoRatio (Institute of Biomedical Technology, University of Tampere). Additionally, double IHC for α-SMA (pericytes) and CD105 (endothelial cells) was performed. In brief, tissue slices were deparaffinized in xylene, hydrated in ethanol and unmasked by citrate buffer pH 6 at 95°C for 20′. Endogenous peroxidase was blocked by 3% H2O2 in PBS and mouse IgG by M.O.M. basic kit (BMK-2202, Vector Lab.). Slides were incubated with α-SMA antibody (DAKO M0851, monoclonal mouse anti-human/rat) 1/200 in MOM diluent at room temperature for 30′. MOM Biotinylated Anti-Mouse IgG reagent was applied for 10 min at room temperature. ABC kit (Vectastain Vector & PK-6100) was added and then stained with DAB. Afterwards, a blocking buffer (10% NRS, 1% BSA, and 0.2% Tween 20 in PBS) was applied for 1 h. Slides were incubated overnight with Ab goat anti-mouse Endoglin (CD105 R&D systems ref. AF1320) 1/50 in PBS + 1/10 blocking buffer. LSAB+ System-AP kit (DAKO K0678) was used according to manufacturer's instructions and Fuchsin+ chromogen was added for 1′ at room temperature. Finally, slides were counterstained with haematoxylin. Microvascular density (CD105+ vessels) and pericyte coverage (CD105+ & α-SMA+ vessels) were assessed by hot spot analysis (up to 6/slide) using Fiji [36].

#### Platinum measurements

A New Wave Research UP193HE ArF* excimer-based laser ablation system (New Wave Research, CA, USA) coupled to an Element XR (Thermo Scientific, Braunschweig, Germany) double-focusing sector field ICP-MS instrument was used to determine the distribution of Pt within thin section of tissues (20 μm paraffinated slices). This LA-unit was equipped with a standard circular ablation cell with a diameter of 5 cm. Thin sections of tissue were scanned with a laser beam with a diameter of 80 μm. The scanning parameters were selected such as to provide square pixels in the final elemental map (80 × 80 μm). The material released from the surface of the sample upon ablation was transported using He as a carrier gas into the ICP ion source. Post-ablation, the He flow carrying the dry sample aerosol was admixed with Ar prior to introduction into the ICP. Both laser beam diameter and laser repetition frequency have been selected carefully, taking into account the nature of the sample and the concentration of the target element. Also, the incident laser fluence has been optimized. Color maps were created of Pt and P distribution and of the Pt/P ratio. LA-ICP-MS settings are summarized in Supplementary Table S1. Pt/P maps were analyzed using Fiji by identifying the peritoneal border region (1.68 mm thickness) and calculating average Pt/P ratio intensity values.

### Tumor growth delay

#### Treatment

Mice were divided into five groups of four animals. Four groups received pretreatment as described previously, while the fifth, Sham, received either daily oral gavage of 0.15 mL PBS or IP injections of 0.15 mL saline solution on day 10 and 14, and underwent intraperitoneal perfusion with saline (without Oxaliplatin). IPC was performed as described previously on day 15 and mice were euthanized on day 28.

#### MR acquisition and image analysis

MR images were acquired at three time points (day 14, 19, and 26) on a 7T system (Bruker PharmaScan 70/16, Ettlingen, Germany) with a mouse body volume coil. Mice were anaesthetized with isoflurane (5% induction, 1.5% maintenance, 0.3 L/min) and warmed with a water-based heating blanket. Anatomical information was obtained with a T2-weighted sequence (TurboRARE) with the following parameters: TR 3661 ms, TE 37.1 ms, 100 μm in-plane resolution, 30 contiguous slices of 600 μm, and acquisition time 9′1”. Pre- and post-contrast T1-weighted images were obtained with a RARE sequence with parameters TR 1464 ms, TE 9.1 ms, 120 μm in-plane resolution, 30 contiguous slices of 600 μm, and acquisition time 4′17”. Dynamic contrast-enhanced MR images (DCE-MRI) were acquired for a single slice using a FLASH sequence with the following parameters: TR 12 ms, TE 3.4 ms, 268 μm in-plane resolution, 550 repetitions, temporal resolution 1.344′', and acquisition time 12′19”. The oblique slice was rotated such that both tumors were covered. The gadolinium-based contrast agent (Vistarem 50 mmol/kg, Guerbet, Villepinte, France) was intravenously injected 1′ after start of the acquisition. Total acquisition time per session was 35′. Tumor volume was measured slice-by-slice using Fiji by multiplying total surface area with voxel thickness (0.6 mm). Relative tumor volume was set as the measured volume divided by the initial volume (day 14). Dynamic time series were analyzed with MIStar version 3.2.62.03 (Apollo MIT, Melbourne, Australia) using curve analysis. ROI of tumors were manually selected and mean values were determined.

### Statistical analysis

Statistical analysis was performed using Graphpad Prism 6 (Graphpad Software, Inc.; La Jolla, CA, USA). Data was analyzed using non-parametric tests (Mann-Whitney-U, Kruskall-Wallis, Dunn's method). Tumor growth delay and MTT IC50 were determined using non-linear regression analysis (exponential growth and dose-response inhibition). Alpha was set at 0.05 for all tests.

## SUPPLEMENTARY TABLE AND FIGURE



## References

[1] Kuijpers AM, Mirck B, Aalbers AG, Nienhuijs SW, de Hingh IH, Wiezer MJ, van Ramshorst B, van Ginkel RJ, Havenga K, Bremers AJ, de Wilt JH, Te Velde EA, Verwaal VJ (2013). Cytoreduction and HIPEC in The Netherlands: Nationwide Long-term Outcome Following the Dutch Protocol. Annals of surgical oncology.

[2] Verwaal VJ, van Ruth S, de Bree E, van Sloothen GW, van Tinteren H, Boot H, Zoetmulder FA (2003). Randomized trial of cytoreduction and hyperthermic intraperitoneal chemotherapy versus systemic chemotherapy and palliative surgery in patients with peritoneal carcinomatosis of colorectal cancer. Journal of clinical oncology : official journal of the American Society of Clinical Oncology.

[3] Verwaal VJ, Bruin S, Boot H, van Slooten G, van Tinteren H (2008). 8-year follow-up of randomized trial: cytoreduction and hyperthermic intraperitoneal chemotherapy versus systemic chemotherapy in patients with peritoneal carcinomatosis of colorectal cancer. Annals of surgical oncology.

[4] Ceelen WP, Flessner MF (2010). Intraperitoneal therapy for peritoneal tumors: biophysics and clinical evidence. Nature reviews Clinical oncology.

[5] Hasovits C, Clarke S (2012). Pharmacokinetics and pharmacodynamics of intraperitoneal cancer chemotherapeutics. Clinical pharmacokinetics.

[6] Heldin CH, Rubin K, Pietras K, Ostman A (2004). High interstitial fluid pressure - an obstacle in cancer therapy. Nature reviews Cancer.

[7] Jain RK, Tong RT, Munn LL (2007). Effect of vascular normalization by antiangiogenic therapy on interstitial hypertension, peritumor edema, and lymphatic metastasis: insights from a mathematical model. Cancer research.

[8] Mehta AM, Van den Hoven JM, Rosing H, Hillebrand MJ, Nuijen B, Huitema AD, Beijnen JH, Verwaal VJ (2015). Stability of oxaliplatin in chloride-containing carrier solutions used in hyperthermic intraperitoneal chemotherapy. International journal of pharmaceutics.

[9] Jerremalm E, Hedeland M, Wallin I, Bondesson U, Ehrsson H (2004). Oxaliplatin degradation in the presence of chloride: identification and cytotoxicity of the monochloro monooxalato complex. Pharmaceutical research.

[10] Willett CG, Duda DG, di Tomaso E, Boucher Y, Ancukiewicz M, Sahani DV, Lahdenranta J, Chung DC, Fischman AJ, Lauwers GY, Shellito P, Czito BG, Wong TZ, Paulson E, Poleski M, Vujaskovic Z (2009). Efficacy, safety, and biomarkers of neoadjuvant bevacizumab, radiation therapy, and fluorouracil in rectal cancer: a multidisciplinary phase II study. Journal of clinical oncology : official journal of the American Society of Clinical Oncology.

[11] Jacquet P, Sugarbaker PH (1996). Clinical research methodologies in diagnosis and staging of patients with peritoneal carcinomatosis. Cancer treatment and research.

[12] Pietras K, Ostman A, Sjoquist M, Buchdunger E, Reed RK, Heldin CH, Rubin K (2001). Inhibition of platelet-derived growth factor receptors reduces interstitial hypertension and increases transcapillary transport in tumors. Cancer research.

[13] Fan Y, Du W, He B, Fu F, Yuan L, Wu H, Dai W, Zhang H, Wang X, Wang J, Zhang X, Zhang Q (2013). The reduction of tumor interstitial fluid pressure by liposomal imatinib and its effect on combination therapy with liposomal doxorubicin. Biomaterials.

[14] Fukumura D, Jain RK (2007). Tumor microvasculature and microenvironment: targets for anti-angiogenesis and normalization. Microvascular research.

[15] Gremonprez F, Willaert W, Ceelen W (2013). Intraperitoneal chemotherapy (IPC) for peritoneal carcinomatosis: Review of animal models. Journal of surgical oncology.

[16] Jain RK (2013). Normalizing tumor microenvironment to treat cancer: bench to bedside to biomarkers. Journal of clinical oncology : official journal of the American Society of Clinical Oncology.

[17] Willett CG, Boucher Y, di Tomaso E, Duda DG, Munn LL, Tong RT, Chung DC, Sahani DV, Kalva SP, Kozin SV, Mino M, Cohen KS, Scadden DT, Hartford AC, Fischman AJ, Clark JW (2004). Direct evidence that the VEGF-specific antibody bevacizumab has antivascular effects in human rectal cancer. Nature medicine.

[18] Barnes SL, Whisenant JG, Loveless ME, Yankeelov TE (2012). Practical dynamic contrast enhanced MRI in small animal models of cancer: data acquisition, data analysis, and interpretation. Pharmaceutics.

[19] Tailor TD, Hanna G, Yarmolenko PS, Dreher MR, Betof AS, Nixon AB, Spasojevic I, Dewhirst MW (2010). Effect of pazopanib on tumor microenvironment and liposome delivery. Molecular cancer therapeutics.

[20] Chatterjee S, Wieczorek C, Schottle J, Siobal M, Hinze Y, Franz T, Florin A, Adamczak J, Heukamp LC, Neumaier B, Ullrich RT (2014). Transient antiangiogenic treatment improves delivery of cytotoxic compounds and therapeutic outcome in lung cancer. Cancer research.

[21] Yu DC, Waby JS, Chirakkal H, Staton CA, Corfe BM (2010). Butyrate suppresses expression of neuropilin I in colorectal cell lines through inhibition of Sp1 transactivation. Molecular cancer.

[22] Jain RK (2014). Antiangiogenesis strategies revisited: from starving tumors to alleviating hypoxia. Cancer cell.

[23] Wolff NC, Randle DE, Egorin MJ, Minna JD, Ilaria RL (2004). Imatinib mesylate efficiently achieves therapeutic intratumor concentrations *in vivo* but has limited activity in a xenograft model of small cell lung cancer. Clinical cancer research : an official journal of the American Association for Cancer Research.

[24] Heldin C-H (2013). Targeting the PDGF signaling pathway in tumor treatment. Cell Communication and Signaling.

[25] Klaver YL, Hendriks T, Lomme RM, Rutten HJ, Bleichrodt RP, de Hingh IH (2010). Intraoperative hyperthermic intraperitoneal chemotherapy after cytoreductive surgery for peritoneal carcinomatosis in an experimental model. The British journal of surgery.

[26] Jimenez W, Sardi A, Nieroda C, Sittig M, Milovanov V, Nunez M, Aydin N, Gushchin V (2014). Predictive and prognostic survival factors in peritoneal carcinomatosis from appendiceal cancer after cytoreductive surgery with hyperthermic intraperitoneal chemotherapy. Annals of surgical oncology.

[27] Giantonio BJ, Catalano PJ, Meropol NJ, O'Dwyer PJ, Mitchell EP, Alberts SR, Schwartz MA, Benson AB (2007). Bevacizumab in combination with oxaliplatin, fluorouracil, and leucovorin (FOLFOX4) for previously treated metastatic colorectal cancer: results from the Eastern Cooperative Oncology Group Study E3200. Journal of clinical oncology : official journal of the American Society of Clinical Oncology.

[28] Hackl C, Man S, Francia G, Milsom C, Xu P, Kerbel RS (2013). Metronomic oral topotecan prolongs survival and reduces liver metastasis in improved preclinical orthotopic and adjuvant therapy colon cancer models. Gut.

[29] Nasti G, Piccirillo MC, Izzo F, Ottaiano A, Albino V, Delrio P, Romano C, Giordano P, Lastoria S, Caraco C, de Lutio di Castelguidone E, Palaia R, Daniele G, Aloj L, Romano G, Iaffaioli RV (2013). Neoadjuvant FOLFIRI+bevacizumab in patients with resectable liver metastases from colorectal cancer: a phase 2 trial. British journal of cancer.

[30] Tamandl D, Gruenberger B, Klinger M, Herberger B, Kaczirek K, Fleischmann E, Gruenberger T (2010). Liver resection remains a safe procedure after neoadjuvant chemotherapy including bevacizumab: a case-controlled study. Annals of surgery.

[31] Starlinger P, Alidzanovic L, Schauer D, Maier T, Nemeth C, Perisanidis B, Tamandl D, Gruenberger B, Gruenberger T, Brostjan C (2012). Neoadjuvant bevacizumab persistently inactivates VEGF at the time of surgery despite preoperative cessation. British journal of cancer.

[32] Liedert B, Pluim D, Schellens J, Thomale J (2006). Adduct-specific monoclonal antibodies for the measurement of cisplatin-induced DNA lesions in individual cell nuclei. Nucleic acids research.

[33] De Smet L, Colin P, Ceelen W, Bracke M, Van Bocxlaer J, Remon JP, Vervaet C (2012). Development of a nanocrystalline Paclitaxel formulation for HIPEC treatment. Pharmaceutical research.

[34] Ozerdem U (2009). Measuring interstitial fluid pressure with fiberoptic pressure transducers. Microvascular research.

[35] Ceelen W, Smeets P, Backes W, Van Damme N, Boterberg T, Demetter P, Bouckenooghe I, De Visschere M, Peeters M, Pattyn P (2006). Noninvasive monitoring of radiotherapy-induced microvascular changes using dynamic contrast enhanced magnetic resonance imaging (DCE-MRI) in a colorectal tumor model. International journal of radiation oncology, biology, physics.

[36] Schindelin J, Arganda-Carreras I, Frise E, Kaynig V, Longair M, Pietzsch T, Preibisch S, Rueden C, Saalfeld S, Schmid B, Tinevez J-Y, White DJ, Hartenstein V, Eliceiri K, Tomancak P, Cardona A (2012). Fiji: an open-source platform for biological-image analysis. Nat Meth.

