# Supplementary material for: Pretreatment with VEGF(R)-inhibitors reduces interstitial fluid pressure, increases intraperitoneal chemotherapy drug penetration, and impedes tumor growth in a mouse colorectal carcinomatosis model
Source: Oncotarget. 2015 Sep 7;6(30):29889–900. doi: 10.18632/oncotarget.5092 (PMC4745770; doi:10.18632/oncotarget.5092)
Supplement: Supplementary file 1 [file oncotarget-06-29889-s001.pdf]

SUPPLEMENTARY TABLE AND FIGURE

Supplementary Table S1: Instrument settings and data acquisition parameters for LA-ICP-MS

Laser ablation	New Wave Research
Type	UP193HE<br>ArF* excimer based laser
Measurements	Mapping
Wavelength, nm	193
Lateral scanning speed, µm/s	80
Repetition frequency, Hz	10
Laser energy, J/cm^2	0.7–0.8
Diameter of laser beam, µm	80
Ablation chamber	Standard cell
ICP-SFMS	Element XR
Mass resolution, m/Δm	300
RF power, W	850
Carrier gas flow rate, L/min	He, 0.5
Make up gas flow rate,(L/min)	Ar, 0.7
Number of runs	200–300
Number of passes	1
Time per pass, s	1

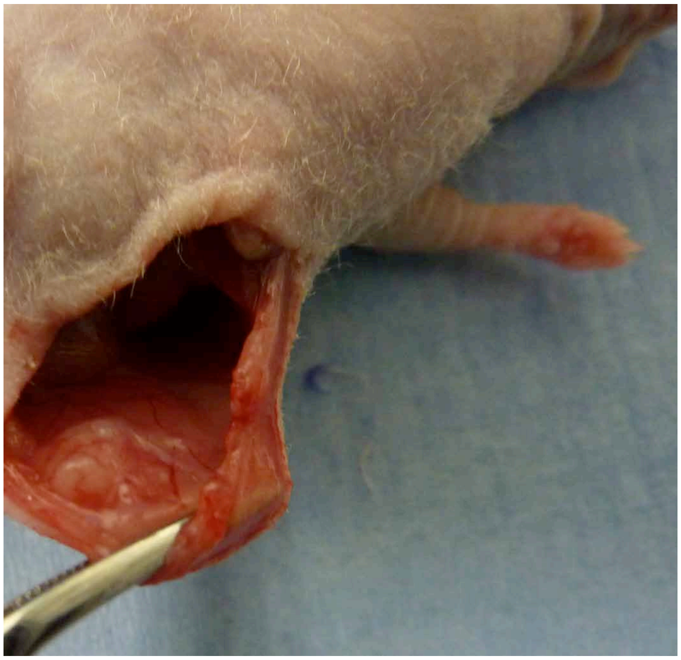

Supplementary Figure S1: Picture of a peritoneal tumor nodule.
